# Supplementary material for: Content-rich biological network constructed by mining PubMed abstracts
Source: BMC Bioinformatics. 2004 Oct 8;5:147. doi: 10.1186/1471-2105-5-147 (PMC528731; doi:10.1186/1471-2105-5-147)
Supplement: Additional File 2 — The original results of the above study (non-essential files are deleted to keep the file size under the limit set by BMC bioinformatics). [file 1471-2105-5-147-S2.bz2 › chilibotAdditionalFile2/dip10/278ID2497053E1101/html/JUN_FOSL1.html]

 


 **JUN** and **FOSL1** 
  
Found 404 abstracts in PubMed, retrieved 10.  
 

 What does Google say? 
 PDF only 
| .edu only 

---

**Interactive relationship** (e.g. stimulation, inhibition, etc)

**Inhibitory relationship**- From transient transfection analysis c  **Jun**  was found to strongly activate a CYP2J2 luciferase reporter construct, but cotransfection with plasmids encoding c Fos or c Fos related antigens Fra 1  [ **FOSL1** ]  and 2, abrogated reporter activity.  Ref: 12737630 Biochem J< MedlineTA>Biochem J,
**Neutral relationship**- Here, the major cis regulatory elements that control IFN gamma inducible NOS2 gene transcription in human lung epithelial cells are identified as composite response elements that bind signal transducer and activator of transcription 1 STAT 1 and activator protein 1  [ **JUN** ]  AP 1  [ **JUN** ] , which is comprised of c Fos, Fra  [ **FOSL1** ]  2, c  **Jun** , and JunD.  Ref: 12788789 Am J Physiol Lung Cell Mol Physiol, 2003
- The oncogene dependent transcriptional activation involves the cooperation between both Raf dependent and Raf independent pathways and is mediated by an AP 1  [ **JUN** ]  site within the fra 1  [ **FOSL1** ]  first intron, which becomes stably occupied by a transcriptionally active Fra 1  [ **FOSL1** ]  containing complex in ras transformed cells.  Ref: 12773579 Mol Cell Biol, 2003

**Non-interactive relationship** (e.g. studied together, co-existance, homology, etc.)

- Under conditions of high rhodopsin availability during exposure to light, Fra  [ **FOSL1** ]  2 and, to a minor degree, FosB substitute for c Fos and enable light induced AP 1  [ **JUN** ]  activity and thus photoreceptor apoptosis.  Ref: 12766089 Invest Ophthalmol Vis Sci, 2003
- By western blot analysis, AP 1  [ **JUN** ] , such as Fra 1  [ **FOSL1** ] ,  **Jun**  B, and phospho CREB protein levels were increased by LT and translocation of NFkappaB p52 from the cytoplasm to the nucleus was increased.  Ref: 12770614 Life Sci, 2003
- These findings strongly suggest that, in C6 cells, Fra 1  [ **FOSL1** ] ,  **Jun**  B, NFkappaB p50, and NFkappaB p52 appear to be involved in the regulation of iNOS mRNA induced by LT.  Ref: 12770614 Life Sci, 2003
- Electrophoretic mobility shift assay EMSA showed that AP 1  [ **JUN** ]  and NFkappaB DNA binding activities were increased at 6 hr and these AP 1  [ **JUN** ]  and NFkappaB DNA bands increased by LT were super shifted when Fra 1  [ **FOSL1** ] ,  **Jun**  B, or NFkappaB p50 antibody was coincubated.  Ref: 12770614 Life Sci, 2003
- To this end, we studied the kinetics of serum stimulated transcription of c fos, c  **jun** , fra 1  [ **FOSL1** ] , egr 1, and cyclinD1 genes, as well as the effects of sodium butyrate, an inhibitor of histone deacetylase activity, on transcription of these genes in normal REF cells and transformants E1A ras.  Ref: 12804589 Biochem Biophys Res Commun, 2003
- Gene expression for Fos was suppressed at both times, while that for  **Fosl1**  and  **Jun**  was augmented at 12 h and suppressed at 48 h.  Ref: 12875578 Acta Otolaryngol, 2003
- In a Gal4 fusion assay, Pdcd4 specifically inhibited activation of c  **Jun**  and c Fos activation domains, but did not inhibit activation of JunB, JunD, Fra 1  [ **FOSL1** ] , or Fra  [ **FOSL1** ]  2.  Ref: 12802278 Oncogene, 2003
- MATERIAL AND METHODS From cDNA microarray screening of those specimens, several gene clusters, including Nos2 and the transcription factors Fos,  **Fosl1** ,  **Jun**  and Nfkb1, were identified as possible upstream regulators of Na transport protein expression.  Ref: 12875578 Acta Otolaryngol, 2003
- Among several immediate early gene products, only  **Jun**  B and Fra 1  [ **FOSL1** ]  protein levels were increased by LNT, and ginsenoside Rd attenuated  **Jun**  B and Fra 1  [ **FOSL1** ]  protein levels induced by LNT.  Ref: 12785733 Arch Pharm Res, 2003
- Deregulated activation of the IL 6 promoter is for the most part mediated by a combined constitutive activation of the nuclear factor NF kappaB p50 and p65 and the activator protein 1  [ **JUN** ]  AP 1  [ **JUN** ]  JunD and Fra 1  [ **FOSL1** ]  family members as demonstrated by electrophoretic mobility shift assays, site directed mutagenesis, and transfection experiments.  Ref: 12727841 Cancer Res, 2003
- Our results suggest that ginsenoside Rd exerts an inhibitory action against NO production via blocking phosphorylation of ERK, in turn, suppressing immediate early gene products such as  **Jun**  B and Fra 1  [ **FOSL1** ]  in C6 glioma cells.  Ref: 12785733 Arch Pharm Res, 2003
- Our data demonstrate for the first time that a combined aberrant activation of NF kappaB p50 and p65 and AP 1  [ **JUN** ]  JunD and Fra 1  [ **FOSL1** ]  in androgen independent prostate cancer cells results in deregulated IL 6 expression, suggesting a novel potential entry point for therapeutic intervention in prostate cancer.  Ref: 12727841 Cancer Res, 2003
